# Real-world experience of emicizumab prophylaxis in young children with hemophilia A: retrospective data from China

**DOI:** 10.3389/fped.2022.992267

**Published:** 2022-10-19

**Authors:** Guoqing Liu, Kun Huang, Gang Li, Yingzi Zhen, Zhengping Li, Zhenping Chen, Runhui Wu

**Affiliations:** ^1^ Hematology Center, Beijing Children's Hospital, Capital Medical University, National Center for Children's Health, Beijing, China; ^2^ Hematologic Disease Laboratory, Hematology Center, Beijing Pediatric Research Institute, Beijing Children's Hospital, Capital Medical University, National Center for Children's Healt, Beijingh, China

**Keywords:** emicizumab, hemophilia a, pediatrics, prophylaxis, bleeds

## Abstract

**Background:**

As a new non-factor therapy for hemophilia A (HA), real-world study of emicizumab is still scarce. This study aimed to investigate the real-world use of emicizumab in Chinese boys with HA.

**Methods:**

Patients with moderate or severe HA were enrolled at Beijing Children's Hospital. They take emicizumab weekly (3 mg/kg) for a month and then went into a maintenance period with a different dosing regimen. After obtaining platelet-poor plasma at end of the loading period and during the maintenance period, coagulation ability and FVIII inhibitor were determined using human and bovine chromogenic Bethesda assay. Patients' bleeding rates were calculated through patients' records from 24 weeks before to at least 6 months after the switch (to emicizumab).

**Result:**

In total, 13 pediatric patients with HA (severe: moderate = 11:2) were enrolled in this study. The patients' age was 3.51 (0.73–6.65) years. Eight had FVIII inhibitors at enrollment and one of them developed FVIII inhibitors again during the switch. The coagulation level of the maintenance period was 19.6 (13.5–32.8) IU/dL (*N* = 10). The median dose of each emicizumab injection was 2.7 (1.3–3.8) mg/kg and the monthly consumption of emicizumab was 5.2 (3.2–6.8) mg/kg/month. After switching to emicizumab, reduced annualized bleeding rate (ABR) [0.5 (0–4) vs. 4 (0–18), *P* < 0.01], annualized joint bleeding rate (AJBR) [0 (0–1.1) vs. 1.0 (0–12), *P* < 0.01], and annualized spontaneous bleeding rate (ASBR) [0 (0–1) vs. 2.0 (0–18), *P* < 0.01] were observed. In patients with or without FVIII inhibitor, similar ABR [0.33 (0–4) vs. 0.5 (0–3), *P* = 0.78], AJBR [0 (0–1.1) vs. 0 (0–0.5), *P* = 0.63], and ASBR [0 (0–1) vs. 0 (0–1.5), *P* = 0.73] were also noticed. Five inhibitor-positive patients (at enrollment) all had their inhibitor titer reduced. In addition, all target joints vanished after switching to emicizumab.

**Conclusion:**

Emicizumab could reduce bleeds in pediatric patients with/without FVIII inhibitors and eliminate target joints.

## Introduction

Hemophilia A (HA) is an inherited bleeding disorder caused by the deficiency of coagulation factor VIII (FVIII). Prophylaxis is recommended by the World Federation of Hemophilia as the standard treatment for patients with severe hemophilia or with severe clinical phenotype, especially in young children (1). However, prophylaxis with FVIII concentrates required frequent venous infusion to keep a steady FVIII level, which would be different for pediatric patients and infants. It is also reported that 20%–30% of hemophiliac patients would develop FVIII inhibitors and ineffective FVIII replacement (2). Thus, although the effectiveness of prophylaxis with FVIII concentrates was proved in the last few decades, it does have disadvantages in routine application.

Emicizumab (Hemlibra®, Roche), which is a humanized, bispecific, monoclonal antibody, as one non-factor product, could bridge activated factor IX and factor X, replacing the function of FVIII and restoring hemostasis in HA patients (3). Considering its high bioavailability and long half-life time, emicizumab has the potential to improve patient adherence largely, which attributes to its long-interval and subcutaneous injection. As reported by previous well-designed clinical trials, emicizumab has a strong capacity to reduce bleeds compared with traditional prophylaxis using FVIII concentrates (4–7). Its effectiveness and safety were also proved in a few recent observational studies (8, 9).

However, due to the expensive price and unaffordable cost, real-world data on its application in pediatric patients with hemophilia A is still scarce, especially in developing countries. Emicizumab has been approved to treat HA patients in China since April 2019. Nowadays, the total number of patients using emicizumab for prophylaxis is less than 100 in all of China. This study was conducted to report the real-world data of our thirteen hemophiliac boys taking emicizumab for prophylaxis in our center.

## Materials and methods

### Ethics

The study was approved by the Ethics Committee of Beijing Children's Hospital and conducted according to the Declaration of Helsinki. Written informed consent was obtained from each enrolled patient and their legally authorized guardian(s).

### Study design

This was a retrospective observational real-world study launched in September 2021, gathering the data of HA children who used emicizumab for prophylaxis from Jun 2019 (the first case) to Sep 2021 with the cases which more than 6 months of emicizumab treatment period.

### Patients and emicizumab administration

Pediatric patients with moderate or severe HA were enrolled to receive prophylactic treatment with emicizumab no matter if they had an FVIII inhibitor at enrollment. The cohort consisted of patients who were regularly followed at our center's outpatient clinic. Data included demographics, diagnosis, prior HA history (inhibitor, prophylaxis treatment regimen), and bleeding events (all bleeds, treated bleeds, joint bleeds, traumatic bleeds) from the 6 months prior to emicizumab were collected from the system historical data of our hospital and patient diaries reviewed at clinic visits. They started taking emicizumab for routine prophylactic treatment after the primary clinic visit. After the first 4 weeks of the loading period with a dosage of 3 mg/kg every week, they went into maintenance (recommended dosage as 1.5 mg/kg weekly, 3 mg/kg every 2 weeks, or 6 mg/kg every 4 weeks).

The first four emicizumab injections (dosage: 3 mg/kg) in the loading period were given at the hospital in case of an emergency like an allergic reaction. Regular calls to each family were made by the study team to collect clinical outcomes and adverse events.

### Blood samples and laboratory analysis

Blood samples were collected using vacuum tubes (2 ml) with 0.109 mol/l buffered citrate during the follow-ups at the end of the loading period and during the maintenance period for FVIII inhibitor and emicizumab coagulation activity. Platelet-poor plasma was obtained after 15 min of centrifugation with reactive centrifugal force (RCF) = 2,500 *g* at room temperature (15–25°C) and kept in a −80°C freezer for further tests. To detect FVIII inhibitor, ACL-TOP 700 autoanalyzer (Instrumental Laboratory, Bedford, MA, United States) was used to determine FVIII inhibitor by bovine chromogenic Bethesda assay based on Coamatic® Factor VIII reagent (Instrumental Laboratory, Bedford, MA, United States). The coagulation ability tests were conducted using a Biophen Plasma Calibrator reagent (Hyphen, Neuville, France) and the results were depicted as equivalent FVIII levels (IU/dL).

### Clinical outcomes

Clinical outcomes including bleeding rates (ABR, annualized bleeding rate; AJBR, annualized joint bleeding rate; ASBR, annualized spontaneous bleeding rate), patients' adverse events, and complications were collected by regular phone calls and confirmed by clinical follow-ups. A target joint is defined as a joint having undergone at least three spontaneous joint bleeds over six consecutive months (10).

### Statistical assay

The statistical analysis and figure generation were performed using GraphPad Prism for Mac (Version 9.1). Due to the limited number of participants, normally distributed data were reported as median with range while non-normally distributed data were reported as median with range. Wilcoxon's test was used to evaluate the difference for non-normal data. *P*-values <0.05 were considered statistically significant.

## Results

### Patients' characteristics and baseline data of dosing regimen

In total, 13 pediatric patients with HA (severe:moderate = 11:2) were enrolled in this study. The two boys with moderate hemophilia A had baseline FVIII levels of 1.9 IU/dL (PN-04) and 1.1 IU/dL (PN-10) respectively. The patients' age was 3.51 (0.73–6.65) years. Eight of them (61.5%) were FVIII inhibitor-negative at enrollment, including five patients who receive prophylaxis with FVIII concentrates and three previously untreated patients before switching to emicizumab. In these eight patients, three of them (PN-03, 04, 08) had a history of FVIII inhibitors before their successful immune tolerance treatment. One of them (PN-08) developed an FVIII inhibitor again after exposure to FVIII concentrates for trauma during the maintenance period. In the other five (38.5%) patients with FVIII inhibitor at enrollment [titer: 15.7 (8.6–44.8) BU], two (PN-09, 12) had rFVII on-demand therapy and the other three (PN-10, 11, 13) used prothrombin complex concentrate (PCC) on-demand therapy before emicizumab. In these five patients, PN-10 has an allergic reaction to FVIII concentrates while PN-13 suffered a failure of immune tolerance induction (ITI). The other three declined to have ITI due to the heavy burden on venous punctures.

During the loading period, all the patients strictly took emicizumab with recommended regimen (3 mg/kg weekly). During the maintenance period, according to families' economic affordability, eight patients took emicizumab every 2 weeks while others had different frequencies from every 10 days to nearly every 3 weeks. The median dose of each emicizumab injection was 2.7 mg/kg with a range from 1.3 to 3.8 mg/kg, which equals the monthly consumption of emicizumab as 5.2 (3.2–6.8) mg/kg/month (Table 1).

**Table 1:** Patients' baseline characteristics and therapy details.

Patient number	Age (years)	Baseline FVIII (IU/dL)	Target joints	Inhibitor history	Inhibitor titer at enrollment (BU)	Treatment before emicizumab	Emicizumab frequency	Emicizumab dosage (mg/kg)	Monthly consumption (mg/kg/month)
PN-01	3.40	<1	NO	(−)	<0.6	prophylaxis	Q2W	3	6
PN-02	4.43	<1	YES	(−)	<0.6	prophylaxis	Q10D	2.2	6
PN-03	2.24	<1	NO	(+)	<0.6	prophylaxis	Q18D	3.8	6
PN-04	6.65	1.9	NO	(−)	<0.6	prophylaxis	Q2W	2.7	5.6
PN-05	6.00	<1	YES	(−)	<0.6	prophylaxis	Q2W	3	6
PN-06	1.38	<1	NO	(−)	<0.6	on-demand	Q2W	2.5	5.2
PN-07	0.73	<1	NO	(−)	<0.6	on-demand	Q2W	3.3	6.8
PN-08	4.75	<1	YES	(+)	<0.6	prophylaxis	Q15D	2.7	5.2
PN-09	3.51	<1	YES	(+)	22.9	rFVII on-demand	Q20D	3.2	4.4
PN-10	4.78	1.1	YES	(+)	44.8	PCC on-demand	Q2W	1.6	3.2
PN-11	3.54	<1	NO	(+)	15	PCC on-demand	Q2W	1.9	3.6
PN-12	2.17	<1	NO	(+)	15.7	rFVII on-demand	Q2W	2.5	5.2
PN-13	2.27	<1	NO	(+)	8.6	PCC on-demand	Q10D	1.3	3.6

Q2W, every 2 weeks; Q10D, every 10 days; Q18D, every 18 days; Q15D, every 15 days. (−), negative; (+), positive. BU, Bethesda unit; FVIII, factor VIII.

Data are depicted as numbers. The frequency and dose refer to the emicizumab regimen.

### Coagulation level test and FVIII inhibitor assay

The coagulation level of the loading period (detected right before the fifth injection of emicizumab) in eight patients was 21.79 (16.3–35.3) IU/dL. In the maintenance period, 10 of them had results of coagulation levels of 19.6 (13.5–32.8) IU/dL. At enrollment, five patients were detected with FVIII inhibitor (PN09–PN13), and the titers were 15.7 (8.6–44.8) BU. During the maintenance period, their FVIII inhibitor titer all decreased and two of them (PN-10, PN-11) had their inhibitor eliminated (<0.6 BU). In these inhibitor-free patients, one of them (PN-08) developed an FVIII inhibitor after exposure to FVIII concentrates due to injury and his inhibitor titer was detected by 1.4 and 2.6 BU in two tests within a month (Table 2).

**Table 2:** Patients' coagulation level and inhibitor development process.

Patient number	Coagulation level at loading period (IU/dL)	Coagulation level at maintenance period (IU/dL)	FVIII inhibitor at enrollment (BU)	FVIII inhibitor process
PN-01	24.7	32.8	<0.6	Continuous negative (<0.6 BU)
PN-02	16.3	30.1	<0.6	Continuous negative (<0.6 BU)
PN-03	NA	NA	<0.6	Continuous negative (<0.6 BU)
PN-04	NA	27.9	<0.6	Continuous negative (<0.6 BU)
PN-05	NA	17.6	<0.6	Continuous negative (<0.6 BU)
PN-06	21.3	14.3	<0.6	Continuous negative (<0.6 BU)
PN-07	33.32	NA	<0.6	Continuous negative (<0.6 BU)
PN-08	NA	21.4	<0.6	7th month:1.4 BU; 8th month: 2.6 BU
PN-09	35.3	22.7	22.9	4th week: 28.9 BU; 10th month: 8.2 BU
PN-10	21.5	NA	44.8	5th week: 15.7 BU
PN-11	NA	17.63	15	10th month: 2.9 BU
PN-12	22.08	17.8	15.7	8th month: <0.6 BU
PN-13	20.3	13.5	8.6	4th week: 4.3 BU; 12th month: <0.6 BU

NA, not available; BU, Bethesda unit; FVIII, factor VIII.

The coagulation level at the loading period and maintenance period was depicted as equivalent FVIII level (IU/dL).

### Clinical outcomes during the switch to emicizumab

**Figure 1:**
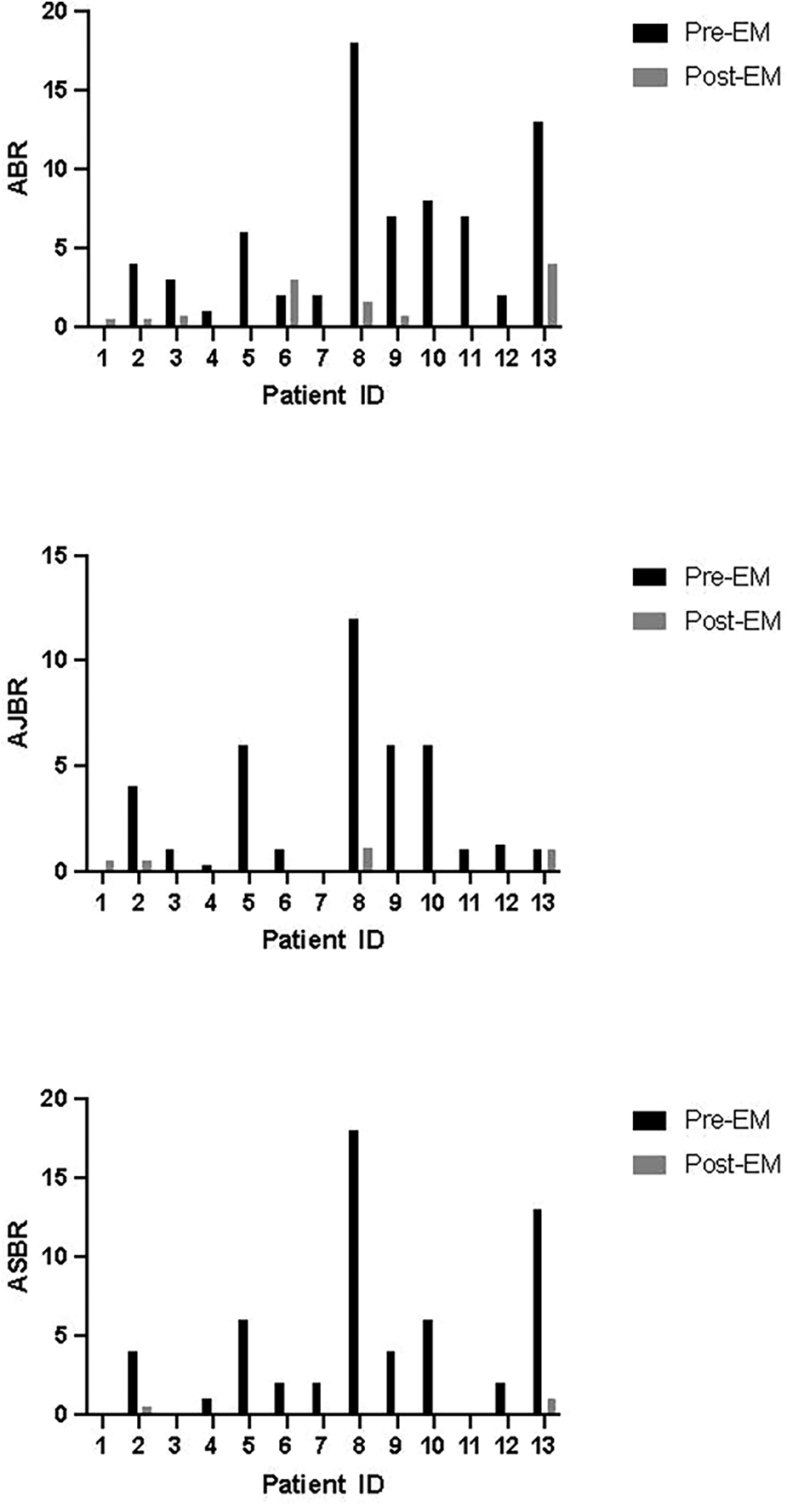
Bleeding rates of each patient before and after switching to emicizumab. ABR, annualized bleeding rate; AJBR, annualized joint bleeding rate; ASBR, annualized spontaneous bleeding rate; EM, emicizumab.

There was a total of 11 bleeding events reported on emicizumab and 6 of them were untreated bleeding events. After switching to emicizumab for an average period of 17.54 (6–26) months, reduced ABR [0.5 (0–4) vs. 4 (0–18), *P* < 0.01], AJBR [0 (0–1.1) vs. 1.0 (0–12), *P* < 0.01] and ASBR [0 (0–1) vs. 2.0 (0–18), *P* < 0.01] were observed compared with the bleeding rates in previous 24 weeks before emicizumab (Figure 1). Moreover, the patients with zero bleeds raised from 7% (*N* = 1) to 46% (*N* = 6) after the switching. Similar coagulation levels [22.05 (14.10–30.78) vs. 19.65 (17.62–29.26), *P* = 0.94] and monthly emicizumab consumption [3.40 (1.38–6.0) vs. 3.53 (2.25–4.79), *P* = 0.84] were observed among patients with or without zero bleeds after switching to emicizumab. The most frequent ABRs were found in PN-06 and PN-13 with coagulation levels of 14.3 and 13.5 IU/dL respectively.

We compared factor VIII prophylaxis before switching to emicizumab vs. emicizumab prophylaxis in six patients. After switching to emicizumab, reduced ABR [0.5 (0–1.6) vs. 3.5 (0–18), *P* = 0.46], were observed compared with the bleeding rates in the previous 24 weeks in six patients who were on factor VIII prophylaxis before emicizumab. And this trend of reduction was also found in AJBR [0.25 (0–1.1) vs. 2.5 (0–12), *P* = 0.75] and ASBR [0 (0–0.5) vs. 2.5 (0–18), *P* = 0.68] although no significant difference was observed.

The bleeding rates of patients with or without FVIII inhibitors were also analyzed. The improved ABR [0.33 (0–4) vs. 7.5 (2–18), *P* < 0.05], AJBR [0 (0–1.1) vs. 3.63 (1–12), *P* < 0.05], and ASBR [0 (0–1) vs. 5 (0–18), *P* < 0.05] were observed in patients with inhibitor (*N* = 6). Reduction of ABR [0.5 (0–3) vs. 2 (0–6), *P* < 0.05], AJBR [0 (0–0.5) vs. 1 (0–6), *P* < 0.05], and ASBR [0 (0–0.5) vs. 2 (0–6), *P* < 0.05] in those patients without inhibitors (*N* = 7) were also noticed. After switching to emicizumab, patients' ABR [0.33 (0–4) vs. 0.5 (0–3), *P* = 0.78], AJBR [0 (0–1.1) vs. 0 (0–0.5), *P* = 0.63], and ASBR [0 (0–1) vs. 0 (0–1.5), *P* = 0.73] were similar among inhibitor-negative patients and inhibitor-positive patients. Compared with those inhibitor-negative ones, patients with FVIII inhibitors showed better improvement in ABR [7.5 (5.26–10.85) vs. 2.0 (−0.5 to 3.5), *P* = 0.01]. This trend of reduction was also found in AJBR [3.63 (0.75–7.23) vs. 1.0 (0–3.5), *P* = 0.19] and ASBR [5.0 (1.5–13.5) vs. 2.0 (0–3.5), *P* = 0.09] although no significant difference was observed.

## Discussion

A long-term analysis that concentrated on HAVEN studies demonstrated that patients using emicizumab had their bleeding rates decline and stabilized at a very low level (11). In this study, more than 94% of target joints finally vanished. Some other studies real-world studies also have observed the extraordinary capacity of bleeding control of emicizumab both in pediatric and adult patients with hemophilia A with a standard dosing regimen (6 mg/kg/month) (3, 9). In our study, the observable improvement of bleeding rates (ABR, AJBR, and ASBR) was also demonstrated in our patients no matter the existence of an FVIII inhibitor in at least 24 weeks of follow-up. In addition, six of them reached the goal of zero bleeding after switching to emicizumab, while their ABRs ranged from 1 to 8 before emicizumab. Also, all five patients who had target joints before switching to emicizumab got rid of target joints with only one joint bleed caused by trauma. This was in accordance with a previous study and indicated that emicizumab has the potential to help pediatric patients with hemophilia A to better protect joints (11).

The patients in our study used different doses and frequencies due to their affordability. Although the recommended dose and frequency were suggested as standard regimens (1.5 mg/kg weekly, 3 mg/kg every 2 weeks, or 6 mg/kg every 4 weeks), final emicizumab consumption ranged from 3.2 to 6.8 mg/kg/month. In our study, the coagulation level and emicizumab monthly consumption were similar in patients with or without zero bleeding rates. This might be partly explained by the effect of injured joints. According to Shima et al., the bleeding rates could be reduced even with low-dose prophylaxis with emicizumab (12). This 5.8-year study reported several cases using emicizumab as low as 0.5–1 mg/kg every week who still received an observable reduction. However, the two patients with low coagulation levels (PN-06 and PN-13) at <15 IU/dL showed higher bleeding rates compared with the others. As reported before, prophylactic treatment with FVIII concentrates must reach a very high trough FVIII level (15 IU/dL) to keep joint bleeding rate to zero, which requests a nearly unreasonably high dose of FVIII concentrate and frequent infusions (13). Although emicizumab showed the advantages of reducing bleeds compared with FVIII concentrates, an inappropriate low-dose regimen should be avoided for better clinical outcomes.

We also analyzed the clinical outcomes of different patients with or without FVIII inhibitors. In our study, all of them gained improved bleeding rates no matter whether they had FVIII inhibitors. This was in accordance with some previous studies like Haven-2 and Haven-4, which found that FVIII inhibitors would not affect the function of emicizumab (5, 6). Also, more observable decrement in bleeding rates was noticed in inhibitor-positive patients compared with those without FVIII inhibitors, which was not hard to understand because of the existence of FVIII inhibitors before switching to emicizumab. To our note, one patient (PN-08) was inhibitor-negative at enrollment, but he was detected with an FVIII inhibitor level of 1.4 BU in April, and it increased to 2.6 BU after 1 month. Considering his FVIII inhibitor history (later got ITI success), the FVIII exposure during his treatment of trauma might be the reason for its recurrence. Another study also reported a similar case which described a boy whose FVIII inhibitor kept negative for more than 2 years and was found to have a high FVIII inhibitor (6BU) after FVIII infusions due to trauma (14). In accordance with previous studies, the inhibitor titer in our patients was reduced as the prophylactic treatment with emicizumab went on, which might attribute to the discontinuation of FVIII concentrates (4).

To our note, a mild elevation of inhibitor titer was observed (from 22.9 to 28.9 BU) at the fifth infusion of emicizumab in PN-08 although his FVIII infusion had been stopped for 5 weeks. Capdevila et al. reported a case with recurrence of FVIII inhibitor although no FVIII product was used (15). Due to the lack of its mechanism, further study should investigate this phenomenon. Considering the potential need for FVIII concentrates like breakthrough bleeds or surgery, patients using emicizumab for prophylaxis are still encouraged to monitor their FVIII inhibitor in routine follow-ups.

In conclusion, we reported real-world data on emicizumab usage in 13 young pediatric patients with hemophilia A in China. Emicizumab could improve bleeds in pediatric patients with or without FVIII inhibitors and help them get rid of target joints. However, implementing standard treatment guarantees a better outcome. These real-world emicizumab reports in this study have some limitations. First, the data included in this study focused on one hospital, more data should be assessed in the future. Second, only 13 children were treated with emicizumab and analyzed in this study. Many unexplained questions need more clinical data to confirm. Additionally, our methods of testing emicizumab activity have not been validated and cannot be used in clinical practice.

## Data Availability

The raw data supporting the conclusions of this article will be made available by the authors, without undue reservation.
